# Evidence Lacking for Endemic Chagas Disease in the United States

**DOI:** 10.3201/eid3205.251840

**Published:** 2026-05

**Authors:** Paul T. Cantey, Marisa Hast, Rebecca J. Chancey, Susan P. Mongomery

**Affiliations:** Centers for Disease Control and Prevention, Atlanta, Georgia, USA

**Keywords:** Chagas disease, *Trypanosoma cruzi*, triatomine, parasites, vector-borne infections

**To the Editor:** The Centers for Disease Control and Prevention Parasitic Diseases Branch (National Center for Emerging and Zoonotic Infectious Diseases, Division of Parasitic Diseases and Malaria) wishes to comment on the Perspective from Beatty et al. (*1*), published last fall. The authors describe infections in the Americas, the presence of multiple vector triatomine insects and *Trypanosoma cruzi* infection in animals in the United States, and the history of locally acquired human cases. Although this article provides supportive information for classifying this pathogen as endemic to the United States, we would like to highlight that human disease caused locally by the pathogen is sporadic, not endemic.

Fewer than 100 locally acquired, vectorborne human *T. cruzi* infections in the country have been documented (*2*). Other documented routes of infection include vertical, transplant-derived, transfusion-derived (before 2007), and occupational exposure-related transmission (*2*,*3*). This number of infections is small compared with the estimated 288,000 persons currently infected in the country (*4*) who acquired the infection elsewhere.

As indicated by the authors, triatomines were identified in the United States in the 1800s and *T. cruzi* was identified in 1916. Data suggest that triatomine species in the United States are primarily sylvatic but occasionally invade homes (*2*). Although high numbers of infected triatomines and mammalian reservoirs have been found in some focal areas, reported human cases do not demonstrate that Chagas disease is emerging in the United States. A combination of triatomine and human factors likely reduces risk. Declaring human Chagas disease endemic could result in universal patient testing that would lead to overtesting of populations with no major risk and the associated costs of false-positive results (for example, healthcare costs, impacts on organ transplantation processes, and unnecessary anxiety for individual patients).

However, continued effort is needed to identify and treat the 288,000 persons with Chagas disease in the United States, including educating healthcare providers to identify high-risk persons and manage the disease. If state partners wish to make Chagas disease nationally notifiable, the Centers for Disease Control and Prevention welcomes the opportunity to work with them to track cases of Chagas disease in the United States. In the meantime, states could voluntarily report Chagas cases using standardized surveillance definitions (https://ndc.services.cdc.gov/case-definitions/chagas).
